# Ultrahigh Charpy impact toughness (~450J) achieved in high strength ferrite/martensite laminated steels

**DOI:** 10.1038/srep41459

**Published:** 2017-02-02

**Authors:** Wenquan Cao, Mingda Zhang, Chongxiang Huang, Shuyang Xiao, Han Dong, Yuqing Weng

**Affiliations:** 1 Special Steel department of Central Iron and Steel Research Institute (CISRI), Beijing 100081, China; 2 School of Aeronautics and Astronautics, Sichuan University, Chengdu 610065, China; 3 School of Materials Science and Engineering, University of Science &Technology of Beijing, Beijing 100083, China

## Abstract

Strength and toughness are a couple of paradox as similar as strength-ductility trade-off in homogenous materials, body-centered-cubic steels in particular. Here we report a simple way to get ultrahigh toughness without sacrificing strength. By simple alloying design and hot rolling the 5Mn3Al steels in ferrite/austenite dual phase temperature region, we obtain a series of ferrite/martensite laminated steels that show up-to 400–450J Charpy V-notch impact energy combined with a tensile strength as high as 1.0–1.2 GPa at room temperature, which is nearly 3–5 times higher than that of conventional low alloy steels at similar strength level. This remarkably enhanced toughness is mainly attributed to the delamination between ferrite and martensite lamellae. The current finding gives us a promising way to produce high strength steel with ultrahigh impact toughness by simple alloying design and hot rolling in industry.

Toughness is an indication of the capacity of a steel to absorb energy and depends on strength as well as ductility significantly. The critical impact toughness is strongly concerned as one of the screening parameters of properties in many steel applications to ensure safety^1^,^2^, such as pipeline steel, bridge steel, car steel, and aeronautic steel, *et al*. With today’s situation of severe environmental pollution and natural resource limitation, the application of high strength steels is increasingly requested to get the weight lightening and safety improvement. However, the impact toughness of widely used low alloy steels (≤8 weight % alloying elements) normally decreases with increasing tensile strength^2^,^3^,^4^,^5^. For instance, the room temperature (RT) impact toughness decreases from ~300–350J at strength level of 0.5 GPa (pipeline steel), to 50–100J at 1.0 GPa (medium carbon steel) and further to only 10–40J at 1.5 GPa (high carbon steel)^6^,^7^,^8^. Generally, the toughness of these low alloy steels is controlled by their inherent resistance of microstructures (grain size, precipitates, etc.) to crack initiation and growth, namely intrinsic toughness mechanism^1^,^5^,^7^. High alloy steels exhibit much higher impact toughness than low alloy steels. For example, the impact energy of high alloyed precipitated hardening steels (austenite steel and nanobainite steel) could be about 200J at strength level of 1.5 GPa and about 50J at 2.0 GPa at RT^9^,^10^,^11^, due to the existence of austenite and addition of Ni and Co. These high alloy steels are much more expensive than low alloy steels, which restricts their application and drives the research and development of low alloy steels with high strength and high toughness. In order to get this target, the dependence of toughness on the strength of low alloy steels has to be altered, i.e., to find a way to evade the dependence of toughness on the intrinsic toughening mechanism in which the impact toughness is controlled by the ductility of constitutive materials.

Remarkably enhanced impact toughness has been reported in composite materials with laminated structure^12^,^13^,^14^,^15^,^16^,^17^,^18^,^19^. Laminate composite consists of different constitutive materials that are alternately separated by discrete interfaces. By roll bonding, much better impact energy than those of constitutive steels can be obtained in laminate composites^15^,^16^. Delamination between layers, a typical extrinsic toughness mechanism^1^,^2^,^12^, was found to play an important role in deflecting crack, imposing new cracks and absorbing energy, which was responsible for high impact toughness and high fracture resistance of laminate composites^12^,^13^,^14^,^15^,^16^,^17^,^18^,^19^. However, the conventional fabrication procedure for laminate composites is very complex, which increases the cost significantly and restricts their manufacturing efficiency in large quantity in industry.

Dual phase steels (DP-steels) consisting of ferrite and martensite can be easily produced in steel industry, which however give impact toughness lower than 150J at strength level of 0.5–0.9 GPa at RT^20^,^21^. It could be expected that if laminated structure with ferrite and martensite is designed in the DP steels, their impact toughness could be improved significantly without sacrificing strength very much. Normally, laminate materials are fabricated by stacking and repeatedly rolling different steels and/or other materials^22^,^23^, which are very complicated and difficult to perform in industry. In addition, the stability of ferrite phase and austenite phase in conventional DP-steels is too low to form a laminated microstructure by hot rolling. Therefore, a redesign of alloying and hot rolling technique is essential to develop laminated structure in new designed DP-steels to fulfill commercial application and scientific research.

In this research, a series of low carbon and low alloy steels alloyed with Mn and Al were designed and fabricated by conventional melting, casting and hot rolling. The ferrite/martensite laminated structure was produced by hot rolling the steel in ferrite and austenite dual phase temperature region and air cooling down to RT. A remarkably enhanced Charpy V-notch impact toughness was demonstrated in these laminate DP steels, 400–450J at strength level of 1.0–1.2 GPa at RT and 400J at −40 °C, when the notch was opened in the rolling plane with direction parallel to the transverse direction. Our results demonstrate a promising and easy pathway towards a new generation of super strong and tough steels for industrial fabrication and application in large quantity.

## Results

### Materials and mechanical properties

**Figure 1:**
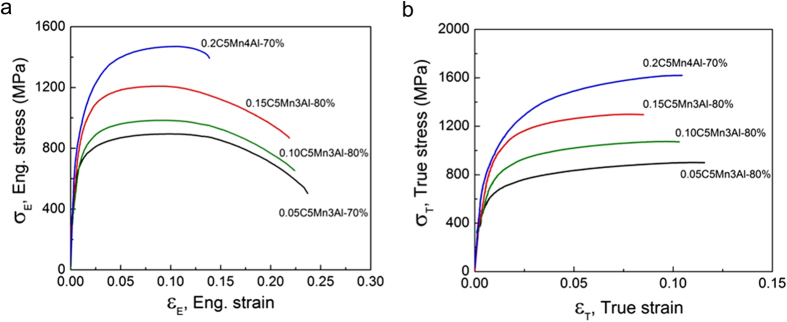
Representative tensile curves of hot rolled FeMnAlC DP-steels. (**a**) Engineering stress-strain curves, (**b**) true stress-strain curves.

We have designed FeMnAlC ferrite/martensite DP-steels with C of 0.05–0.2%, Mn of 5% and Al of 3–4% (weight%, Supplementary Table S1), in which Mn is added to obtain air hardening capacity and Al is to widen the dual phase temperature region. Figure 1(a) shows the engineering stress-strain curves of the laminate steels hot rolled for 70–80% thickness reduction. These steels have tensile strength of 0.81–1.48 GPa and elongation-to-fracture of 12–20%, depending on C content (Supplementary Table S2). Normally, the higher the strength, the lower is the tensile elongation. Besides, these new steels exhibit high work hardening rate, resulting in high uniform elongation of 8–10% (Supplementary Table S2). Figure 1(b) presents the true stress-strain curves. Work hardening behavior is obvious, and the strain hardening exponent for these new designed steel is calculated as 0.13–0.19.

**Figure 2:**
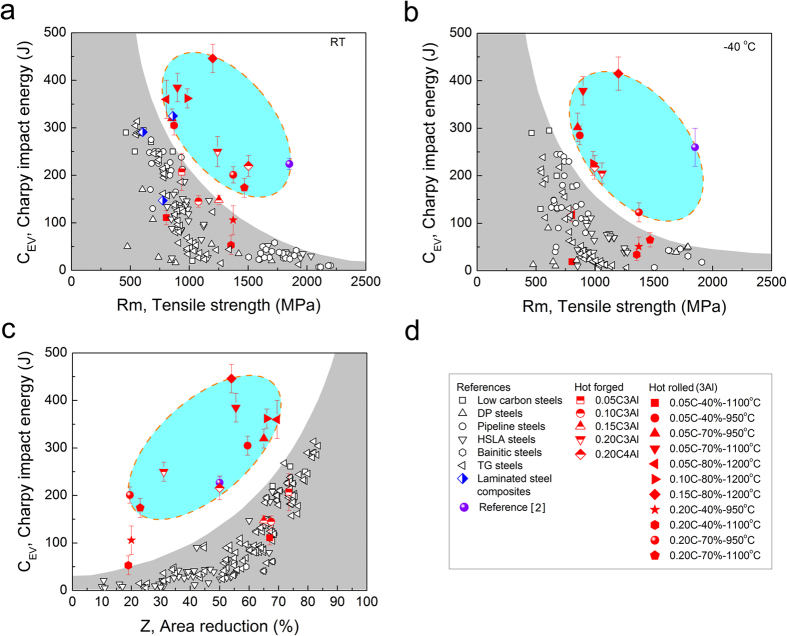
Charp V-notch impact properties of FeMnAlC DP-steels. (**a**) Impact toughness (RT) vs. tensile strength, indicating remarkable enhanced toughness at the same strength level in comparison with other homogeneous low alloy steels; (**b**) Impact toughness (−40 °C) vs. tensile strength; (**c**) Impact toughness (RT) vs. tensile area reduction; (**d**) Description of the symbols. The referenced data from literature include low carbon steels^24^,^25^, DP steels^20^,^21^,^26^,^27^, pipeline steels^28^,^29^,^30^,^31^,^32^,^33^, HSLA steels^2^,^4^,^6^,^34^,^35^,^36^,^37^,^38^, Bainitic steels^39^,^40^,^41^,^42^,^43^, laminated steel composites^13^,^15^,^18^, and our TG steels^44^.

In order to reveal the toughness of our designed steel, standard Charpy V-notch impact samples were prepared. Ultrahigh Charpy V-notch toughness of 300–450J was achieved with strength as high as 0.8–1.2 GPa (seeing Supplementary Tables S2-S3). Such ultrahigh toughness is much excellent than those high toughness steels, such as pipeline steels and high strength low alloy (HSAL) steels (usually <300J) with similar strength^2^,^4^,^6^. We plot the impact energy against tensile strength in Fig. 2(a) by collecting the data of typical low alloy steels and some laminated steel composites from literature. It is clear that for conventional homogenous low alloy steels the impact toughness decreases with an increase in tensile strength, following typical trade-off relation. This typical inverted relation limits the toughness lower than 150J at very high strength level (>1.2 GPa). However, our new designed DP-steels jump out of this traditional exclusive relationship between toughness and strength. Typically, the highest impact toughness of 446J is achieved at tensile strength of 1.2 GPa that is very difficult to obtain in conventional homogenous bcc steels^6^,^7^,^8^. Meanwhile, the steels maintain high toughness of 300–400J even at −40 °C, which is almost 1.5 times higher than that of laminate ultrafine grain-structure steel^2^, as seen in Fig. 2(b). It should be pointed out that the dispersion of current results in Fig. 2 implies that the toughness does not strictly depend on the tensile strength and the area reduction, as well as the yield stress (Supplementary Fig. S3). In fact, the impact toughness is mainly determined by the lamellae density, which will be explained in detail in the discussion section. In the transverse plane the toughness of new designed steel decreases to about 60–120J at RT (Supplementary Table S3), which is much lower than that in the rolling plane but still better than that of conventional DP-steels^20^,^21^.

Figure 2(c) shows that the dependence of toughness on the tensile area reduction values of the new designed steel is clearly different from that of conventional steels. Generally, the higher the area reduction, the higher is the impact toughness, because the impact toughness is mainly controlled by the local plastic deformation capacity of steels. For instance, in conventional steels when the tensile area reduction is about 20% the toughness is usually below 20J, while 60% area reduction corresponds to 60–100J impact toughness. However, in the new designed steels 60% tensile area reduction usually yields more than 300J impact toughness. This large difference indicates that the toughening mechanism of new designed steels is completely different from conventional steels, i.e. weakly depending on the local deformation capacity of steels. Obviously, our experimental result demonstrates that ultrahigh impact toughness can be developed with very low tensile area reduction.

### Laminate microstructures and fractography

**Figure 3:**
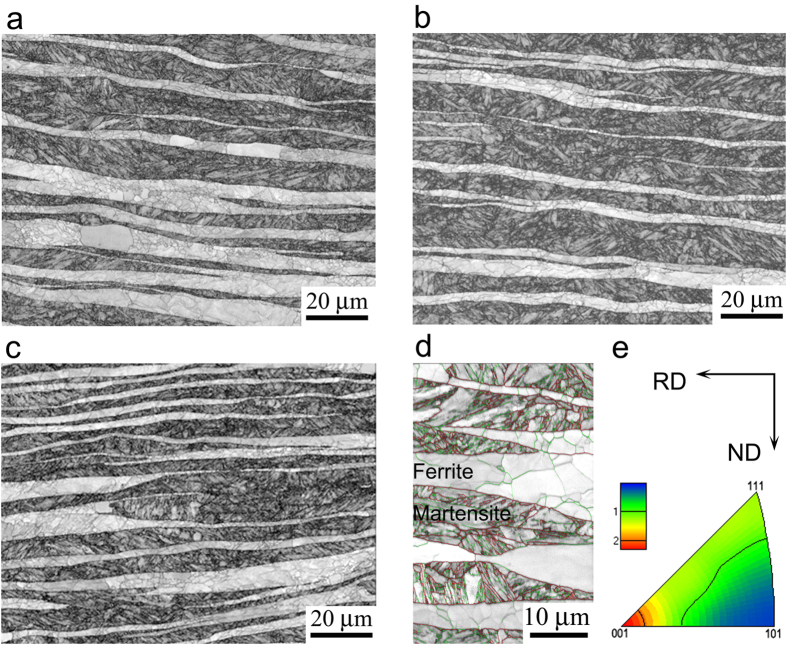
Microstructures of hot rolled *x*C5Mn3Al (*x*: content of C) steels examined by SEM-EBSD. (**a**) 0.05C5Mn3Al, (**b**) 0.10C5Mn3Al, (**c**) 0.15C5Mn3Al, in which the phase with light grey color is ferrite lamella and the dark is martensite lamella. (**d**) EBSD micrograph at high magnification, in which the high-angle boundaries are marked with red lines and the low-angle boundaries are marked with green lines. (**e**) Inverse pole figure for the rolling direction, showing weak <110> deformation texture.

The microsturctural examinations verify that laminated structure is produced in the new DP steels after hot rolling, as shown in Fig. 3(a–d). The laminated structure is characterized by ferrite and martensite alternately stacked in the specimen thickness direction (ND in Fig. 3(e)). The ferrite lamellae consist of dislocation cells and subgrains, while the martensite lamellae are composed of many ultrafine grains and thin martensite lathes, as shown in Fig. 3(d). The microhardness for martensite and ferrite is about 425–660 Hv and 185–205 Hv, respectively, according to their C content. Such soft/hard stacking structure is verified to be both strong and tough^1^,^22^,^23^,^45^. The thickness of ferrite/martensite lamellae is dependent on C content, rolling temperature and rolling strain, but the length is usually in several millimeter (seeing Supplementary Fig. S4). Texture analysis indicates a weak <110> deformation texture parallel to the rolling direction, as shown in Fig. 3(e). Based on the phase diagram of 5Mn3Al steel, the microstructure is composed of ferrite and austenite phase at dual phase temperature range (900–1250 °C), and both of them can be flattened by hot rolling with large thickness reduction. After air cooling down to RT, the already flattened ferrite grains remain to form ferrite lamellae, while the flattened austenite grains became martensite lamellae by a phase transformation.

**Figure 4:**
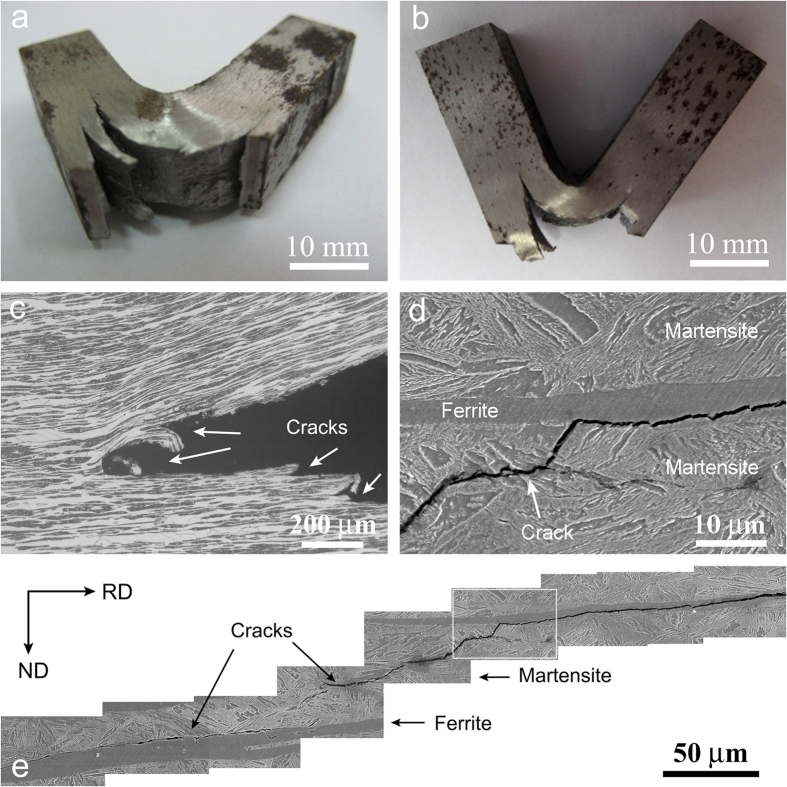
The fractography of the V-notch Charpy impact specimens. (**a**) 0.15C5Mn3Al with the highest impact energy of 446J, (**b**) 0.05C5Mn3Al with impact energy of 360J, (**c**) cracks tearing laminates, (**d**) an enlarge view of (**e**), (**e**) crack propagating in both ferrite and martensite, as well as theirs interfaces.

When impacted from thickness direction (ND in Fig. 3(e)), the alternately stacked ferrite/martensite can significanlty deflect and blunt the crack propogation. Figure 4(a,b) are the typical optical images of impacted samples, showing clearly that cracks branched parallel to the rolling direction. The cracks tearing laminates are presented in Fig. 4(c). Close observations show that there are many cracks propagating along the rolling direction in both ferrite and martensite, as well as their interfaces, as indicated by arrows in Fig. 4(c–e). Such delamination between ferrite and martensite lamellae is believed to contribute greatly to the enhancement of impact toughness. The higher the degree of delamination, the higher is the absorption energy.

### Development of ferrite/martensite laminated structure

**Figure 5:**
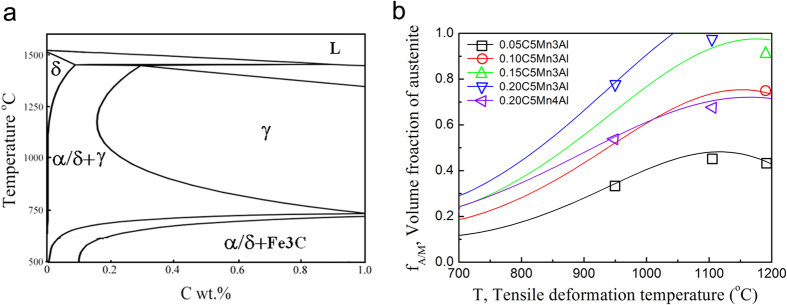
(**a**) Phase diagram of 5Mn3Al steel. (**b**) Austenite volume fraction of 5Mn3Al steels with different C content calculated by Thermal-CAL (solid lines) and measured by optical micrography (open symbols).

The phase diagrams of FeMnAlC steels and phase volume fractions at given composition were calculated by using the Thermo-Calc software with TCFE 7 database. Figure 5(a) shows the phase diagrams for the 5Mn3Al steel. It can be seen that both austenite phase (*γ*, faced-centered cubic, fcc) and ferrite phase (*α*, body-centered cubic, bcc) exist between 900–1250 °C for 5Mn3Al steel when the C content is smaller than 0.2%. Our calculation demonstrates that for 5Mn steel with 3–4% Al it is easy to obtain fcc + bcc (*α *+ *γ*) dual phases at temperature between 900–1250 °C, which is normally the working temperature for thermal-mechanical treatment in steel industry. The phase volume fractions of ferrite and martensite in the hot rolled 5Mn3Al steels are calculated and measured according to the color difference in optical micrographs (Supplementary Fig. S4). Figure 5(b) presents the calculated and measured results, which are in good agreement with each other. This agreement could be understood by the slow transformation of austenite to ferrite that is mainly controlled by the slow partitioning of Mn into austenite and Al into ferrite. At 900–1200 °C, the diffusion coefficients of Mn and Al are 2–4 order of magnitude lower than that of C (Supplementary Fig. S5)^46^. The addition of Mn and Al makes not only the austenite and ferrite dual phase temperature region much broad, but also the two phases very stable at high temperature, which provide an opportunity for rolling the granular austenite and ferrite grains into lamellar shape. During hot rolling, the microstructure evolution of the studied steels could be divided into three steps. Firstly, very large size and granular shaped austenite/ferrite microstructure develops during austenization by means of the portioning of C, Mn and Al elements at 950–1200 °C. Secondly, the granular austenite/ferrite grains are flattened into lamellar shape by the geometric thinning of the prior granular shaped austenite/ferrite coarse grains after hot rolling (large thickness reduction). Finally, the flattened austenite lamellae transform to martensite lathes by air cooling, leading to the formation of ferrite/martensite laminated structure.

## Discussion

We have fabricated ferrite/martensite laminated structure in FeMnAlC steels by simple alloying design and hot rolling, and revealed ultrahigh impact toughness combined with high tensile strength that cannot be realized in conventional homogeneous low alloy steels. There are two questions that should be answered in order to understand why the current laminate steels are both strong and tough. One is why ferrite/martensite laminated structure is seldom found in conventional steels, but can form in current 5Mn3Al DP steel. The second is the dominate mechanism responsible for such high toughness at high strength level, which leads to a break of the traditional strength-toughness exclusive relationship.

The first question is related to the processing temperature and the phase stability of laminated microstructure. For conventional low carbon and low alloy steel, austenization at temperature region of 900–1250 °C usually leads to a single phase (austenite) in the steel. Of cause, a dual phase structure can not be produced at this high temperature region by any hot deformation. It is possible for conventional steels to develop a dual phase laminated structure when the steel is deformed in dual phase region at relative low temperature level of 727–900 °C^47^, at which the deformation stress increases significantly and thus is difficultly realized in modern steel industry. Another reason is that recrystallization and grain growth controlled by the fast diffusion of carbon atoms occur easily in conventional steels during high temperature processing, which increases the opportunity to form a granular microstructure rather than laminated structure. However, in our new designed steels the addition of Mn and Al elements stabilizes austenite and ferrite phases at high temperature due to their sluggish diffusion in steel (Supplementary Fig. S5), which suppresses the growth of lamellae to granular shape. Therefore, it is possible to roll both austenite and ferrite grains simultaneously into very thin lathes by large thickness reduction (Fig. 3 and Supplementary Fig. S4).

**Figure 6:**
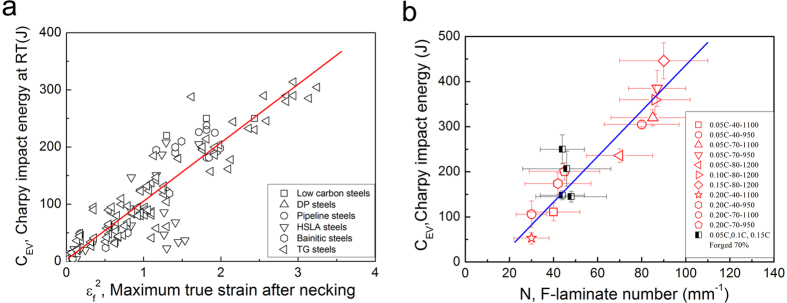
(**a**) The dependence of the V-notch impact energy at RT on the necking strain (*ε*_*f*_); (**b**) Plot of V-notch impact energy against the number (n) of ferrite lamella along thickness direction of our designed steel. The selected data from literature are as same as those in Fig. 2.

The second question is attributed to the unique toughness mechanism in laminate steel. Basically, damage tolerance is related to resistance of materials to crack nucleation and propagation. For most ductile materials, inherent resistance influenced by microstructures is the primary source of fracture toughness that controls crack nucleation and propagation. As shown in Fig. 6(a), the Charpy impact energy depends on the square of necking strain (*ε*_*f*_ = *ln*(*A*_*0*_*/A*), where *A*_*0*_ is the initial sample area and *A* is the fractured sample area for conventional homogenous steels, which suggests that the damage tolerance in these steels is dominated by their local deformation capacity. Higher local plastic deformation capacity enlarges the plastic zone ahead of crack tip, which makes crack nucleation and propagation difficult and thus improve the damage tolerance. Most conventional steels are toughened in this manner. As a consequent, their strength and toughness have to follow the conflicting relation between strength and plasticity^1^,^2^,^5^,^48^, as revealed in Fig. 2(a). However, if the extrinsic toughening mechanisms control fracture, for instance crack deflection, crack blunting and crack bridging, it is expected that the mutually exclusive relation between strength and toughness can be broken^1^,^2^,^12^. Because, the extrinsic toughening mechanisms can significantly reduce the local stress density and/or change the stress state at the crack tip, making crack propagation more difficult^1^,^12^,^49^. Figure 6(b) shows that the Charpy impact energy of current laminate steels depends linearly on the number of ferrite lamella, i.e., the toughness of current steels does not depend on strength and tensile area reduction (Fig. 2 and Supplementary Fig. S3). In laminated materials, when the former lamella was broken the stress condition of the rigid region ahead of the notch tip could be changed from the plane strain state to less detrimental plane stress state, leading to crack propagation along the rolling direction (delaminating lamellae), as shown in Fig. 4. Once delamination occurs between ferrite and marteniste lamellae, a remarkably impact energy is absorbed by both tearing the lamellae/interfaces and renucleating a crack in neighbor lamella. It is possible that a repeated transition between the plane strain state and the plane stress state in the front of crack tip occurs in ferrite/martensite lamellae, which makes the crack propagation much difficult and thus enhance the absorbed energy significantly. It has been revealed that longer delamination length and more number of delamination of interfaces increases the damage tolerance and fracture absorbed energy^2^,^12^,^13^,^14^,^15^,^16^. Therefore, the ideal case is that all lamellae/interfaces delaminate. Such situation is verified in our laminate steels that increasing the numbers of lamellae/interfaces can increase absorbed energy (Fig. 6(b)). This unique toughening mechanism makes the ferrite/martensite laminated steels both strong and tough that cannot be realized in conventional low alloy steels.

In conclusion, an ultrahigh Charpy impact toughness of about 450J at high tensile strength of 1.2 GPa (along the rolling direction) is achieved in a laminated ferrite/martensite steel. This excellent property exceeds the impact toughness of most low alloy steels and steel laminate composites, as seen in Fig. 2(a). An extrinsic toughness mechanism, i.e. delamination of lamellae and interfaces, is demonstrated to control the fracture behavior of current laminate steels, which is responsible for the ultrahigh toughness. By simple alloying design to control the stability of austenite/ferrite phase in the dual phase temperature region, a laminated structure can be produced by hot rolling. The present study shows a promising and easy method towards a new generation of super strong and tough steels for industrial fabrication and application in large quantity.

## Methods

### Material Fabrication

The chemical composition of FeMnAlC steels with C of 0.05–0.2%, Mn of 5% and Al of 3–4% are designed as given in Supplementary Table S1, in which Mn is added to obtain the air hardening capacity, Al is to widen the dual phase region. The steels were prepared by high frequency induction furnace in a vacuum atmosphere and casted into ingots with weight of 50 Kg. Then the ingot was heated up to a temperature of 1200 °C and hot forged into a slab with dimension of 40 × 100 × 200 mm^3^. Thereafter, the slab was homogenized at a given temperature (1200 °C, 1100 °C and 950 °C, respectively) for 2 hours. Finally, the slabs were hot rolled with different thickness reductions (40%, 70% and 80%, respectively) with final rolling temperature of above 800 °C followed by air cooling to room temperature. Detail processing procedure is shown in Supplementary Fig. S1.

### Mechanical Testing

Uniaxial tensile test and Charpy V-notch impact test were conducted by using Instron machine (WDT-10) and tensile machine (WE-300B). The standard dog-bone shaped tensile specimens with gauge length of 30 mm and diameter of 5 mm were cut from the hot rolled slabs with tensile direction parallel to the rolling direction. The uniaxial tensile tests were performed at strain rate of 10^−3^ s^−1^ and at room temperature. Standard specimens for Charpy V-notch impact samples with a dimension of 10 × 10 × 55 mm^3^ and a 45° V groove in the middle of the sample were cut from the hot rolled slabs. In order to reveal the toughness of our designed steel, two kinds of samples were prepared. One was cut from hot rolled slabs with specimen length direction parallel to the rolling direction and the notch was opened in the rolling plane with notch direction parallel to the transverse direction (shortly named as RTT). Another one was also cut from the hot rolled slabs with specimen length direction parallel to the rolling direction but the notch was opened in the transverse plane with notch direction parallel to the normal direction (shortly named as RNN). The sample orientation is schismatically shown in Supplementary Fig. S2. Impact tests were carried out at room temperature and −40 °C.

### Microstructure Characterizations

Optical microscopy, S-4300 field emission SEM and electron-backscattered diffraction (EBSD) in a LEO 1530 field emission SEM were employed for microstructure characterizations. The raw EBSD data was processed by orientation averaging using the VMAP software to reduce orientation noise. Further analysis of these EBSD data was carried out by HKL software (Channel 5). The volume fractions of the phases were measured according to the color difference in the optical micrograph and calculated by using the Thermo-Calc software with TCFE 7 database provided by the CISRI-TCS Joint Open Laboratory.

## Additional Information

**How to cite this article**: Cao, W. *et al*. Ultrahigh Charpy impact toughness (~450J) achieved in high strength ferrite/martensite laminated steels. *Sci. Rep.* **7**, 41459; doi: 10.1038/srep41459 (2017).

**Publisher's note:** Springer Nature remains neutral with regard to jurisdictional claims in published maps and institutional affiliations.

## Supplementary Material

Supplementary Meterials
