# Supplementary material for: Ultrahigh Charpy impact toughness (~450J) achieved in high strength ferrite/martensite laminated steels
Source: Sci Rep. 2017 Feb 2;7:41459. doi: 10.1038/srep41459 (PMC5288709; doi:10.1038/srep41459)

**Supplementary materials**

**Ultrahigh Charpy impact toughness (~450J) achieved in high strength ferrite/martensite laminated steels**

Wenquan Cao^1^, Mingda Zhang^1^, Chongxiang Huang^2^, Shuyang Xiao^3^， Han Dong^1^ & Yuqing Weng^1^

1. Special Steel department of Central Iron and Steel Research Institute (CISRI), Beijing 100081, China.

2. School of Aeronautics and Astronautics, Sichuan University, Chengdu 610065, China

3. School of Materials Science and Engineering, University of Science &Technology of Beijing, Beijing 100083, China.

**Table S1** The chemical compositions of the studied FeMnAlC steels

| Steels | C (wt.%) | Mn (wt.%) | Al (wt.%) | P (ppm) | S (ppm) | N (ppm) |
| --- | --- | --- | --- | --- | --- | --- |
| 0.05C5Mn3Al | 0.05 | 5.0 | 3.0 | <100 | <50 | <50 |
| 0.10C5Mn3Al | 0.10 | 5.0 | 3.0 | <100 | <50 | <50 |
| 0.15C5Mn3Al | 0.15 | 4.92 | 2.75 | <100 | <50 | <50 |
| 0.20C5Mn3Al | 0.20 | 5.0 | 3.0 | <100 | <50 | <50 |
| 0.20C5Mn4Al | 0.20 | 4.78 | 3.92 | <100 | <50 | <50 |

**Table S2** Room temperature mechanical properties of hot rolled plates with 40%, 70% and 80% thickness reduction at different deformation temperature

| **Processing** | **Materials** | **Yield strength (MPa)** | **Ultimate tensile strength (MPa)** | **Uniform elongation (%)** | **Total elongation (%)** | **Area reduction**  **（%）** |
| --- | --- | --- | --- | --- | --- | --- |
| **Hot rolling** | **0.05C3Al-1200-80%** | 517 | 807 | 8.5 | 22.0 | 69.5 |
|  | **0.05C3Al-1100-70%** | 519 | 853 | 9.8 | 22.3 | 65.0 |
|  | **0.05C3Al-950-70%** | 509 | 899 | 10.3 | 22.5 | 65.5 |
|  | **0.05C3Al-1100-40%** | 488 | 805 | 10.8 | 22.8 | 67.0 |
|  | **0.05C3Al-950-40%** | 517 | 872 | 10.3 | 20.3 | 59.5 |
|  | **0.10C3Al-1200-80%** | 645 | 985 | 9.0 | 18.3 | 66.0 |
|  | **0.15C3Al-1200-80%** | 785 | 1199 | 8.0 | 14.8 | 54.0 |
|  | **0.20C4Al-950-70%** | 820 | 1468 | 10.0 | 12.8 | 23.0 |
|  | **0.20C4Al-1100-70%** | 574 | 1374 | 10.3 | 12.0 | 19.5 |
|  | **0.20C4Al-950-40%** | 690 | 1355 | 9.5 | 11.0 | 19.0 |
|  | **0.20C4Al-1100-40%** | 586 | 1373 | 10.8 | 13.8 | 20.0 |
| **Hot forging** | **0.05C3Al-1200-65%** | 613 | 936 | 8.7 | 19.5 | 73.5 |
|  | **0.10C3Al-1200-65%** | 739 | 1078 | 8.0 | 16.3 | 67.5 |
|  | **0.15C3Al-1200-65%** | 867 | 1252 | 7.8 | 15.0 | 65.0 |
|  | **0.20C3Al-1200-65%** | 998 | 1240 | 7.0 | 12.5 | 50.0 |
|  | **0.20C4Al-1200-65%** | 1060 | 1503 | 3.5 | 8.0 | 31.0 |

**Table S3** Charpy-V-notch impact properties and average number of ferrite lamella along thickness direction of the studied DP-steels.

|  | Steel | | Austenization Temperature (^o^C) | Thickness reduction (%) | | Charpy V-notch impact energy (KV2)  at 25^o^C(J) | | | Charpy V-notch impact energy (KV2)  at -40^o^C (J) | | | Average number of Ferrite-Lamina  along thickness (1/mm) |
| --- | --- | --- | --- | --- | --- | --- | --- | --- | --- | --- | --- | --- |
|  |  |  |  |  |  | RTT | | RNN | RTT | | RNN |  |
|  |  | | | | | | | | | | | |
| **Hot rolling** | **0.05C3Al** | 1200 | | | 80 | 360 | 119.9 | | 118 | 51 | | 85 |
|  | **0.05C3Al** | 1100 | | | 70 | 320 | 101 | | 302 | 60 | | 85 |
|  | **0.05C3Al** | 950 | | | 70 | 385 | 63 | | 379 | 49 | | 87 |
|  | **0.05C3Al** | 1100 | | | 40 | 111 | 81 | | 19 | 16 | | 40 |
|  | **0.05C3Al** | 950 | | | 40 | 305 | 71 | | 285 | 53 | | 80 |
|  | **0.10C3Al** | 1200 | | | 80 | 362 | 97.3 | | 226 | 41 | | 86 |
|  | **0.15C3Al** | 1200 | | | 80 | 446 | 79.5 | | 415 | 57 | | 90 |
|  | **0.20C4Al** | 950 | | | 70 | 106 | 17 | | 65 | 8 | | 45 |
|  | **0.20C4Al** | 1100 | | | 70 | 53 | 11 | | 123 | 12 | | 42 |
|  | **0.20C4Al** | 950 | | | 40 | 106 | 17 | | 34 | 6 | | 30 |
|  | **0.20C4Al** | 1100 | | | 40 | 53 | 11 | | 51 | 9 | | 30 |
|  |  |  | | |  |  |  | |  |  | |  |
| **Hot forging** | **0.05C3Al** | 1200 | | | 65 | 207 | - | | - | - | | 46 |
|  | **0.10C3Al** | 1200 | | | 65 | 145 | - | | - | - | | 48 |
|  | **0.15C3Al** | 1200 | | | 65 | 148 | - | | - | - | | 44 |
|  | **0.20C3Al** | 1200 | | | 65 | 220 | - | | 218 | - | | 50 |
|  | **0.20C4Al** | 1200 | | | 65 | 250 | - | | 206 | - | | 44 |

**Figure S1** Schematic illustration of material preparation.


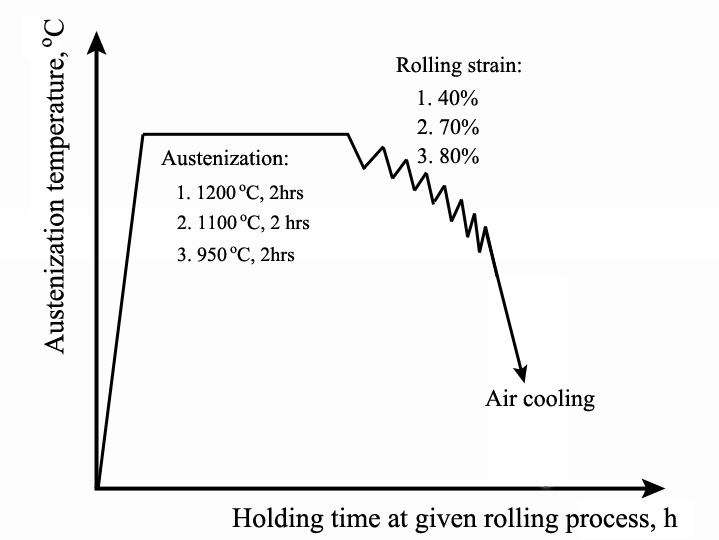


**Figure S2** Schematic diagram of the Charpy V-notch impact test specimens and the tensile specimen.


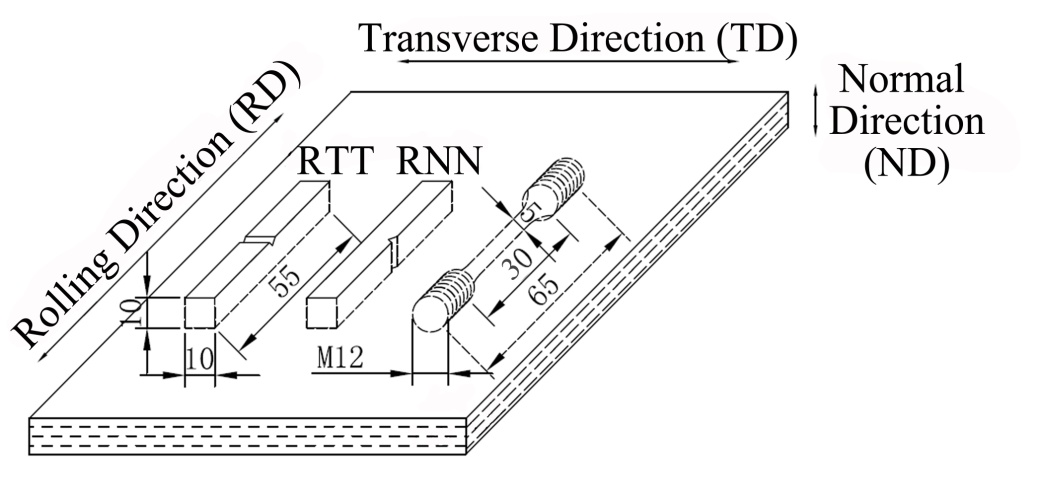


**Figure S3** Charp V-notch impact properties of FeMnAlC DP-steels: (a) Impact toughness (RT) vs. yield strength; (b) Impact toughness (-40^o^C) vs. yield strength. The references and symbols are as same as those in Figure 2 in the text. Basically, increasing the lamella density can lead to a remarkable enhancement of toughness at a yield strength level between 500-1000 MPa, as indicated by the arrow.


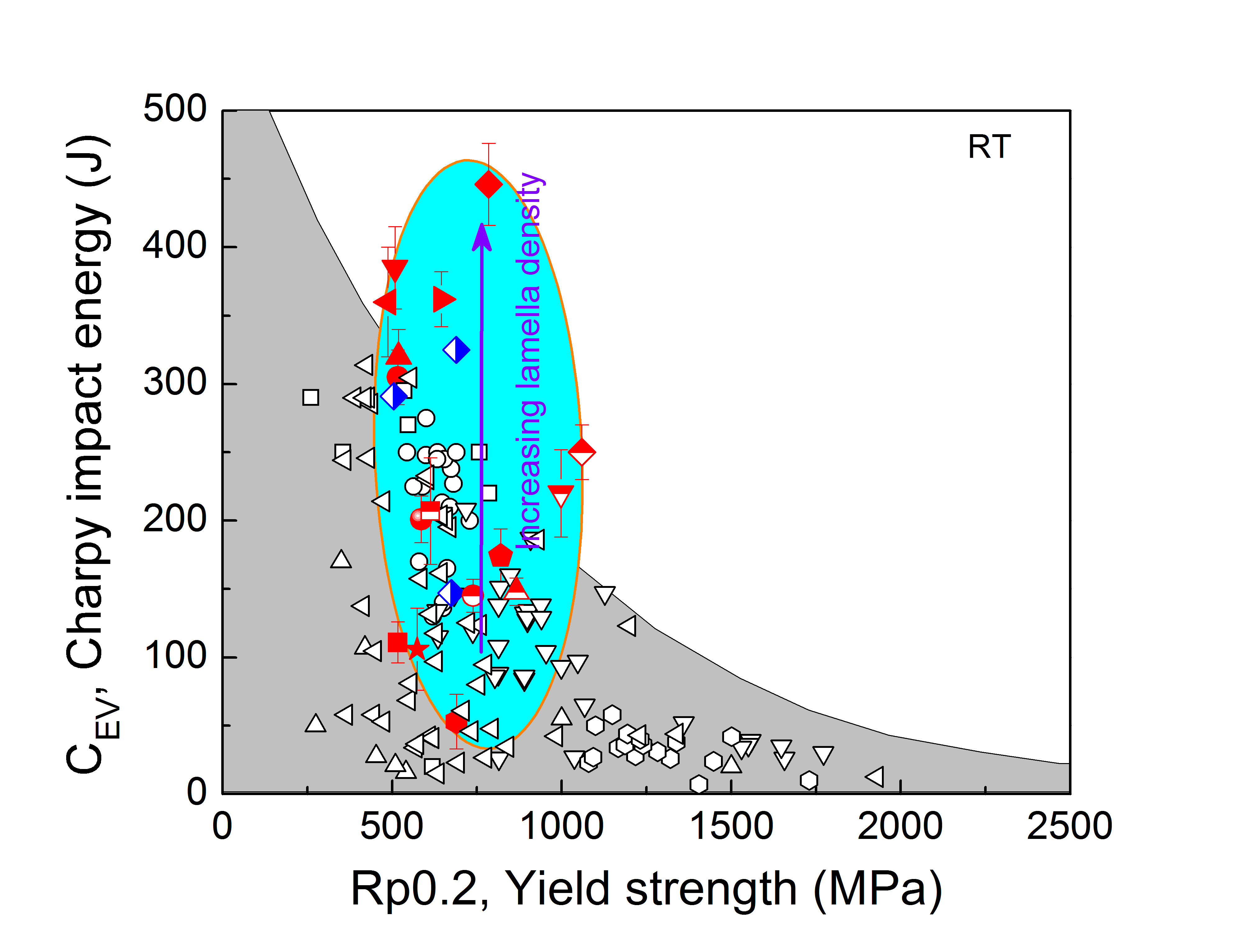


(a)

(b)

**
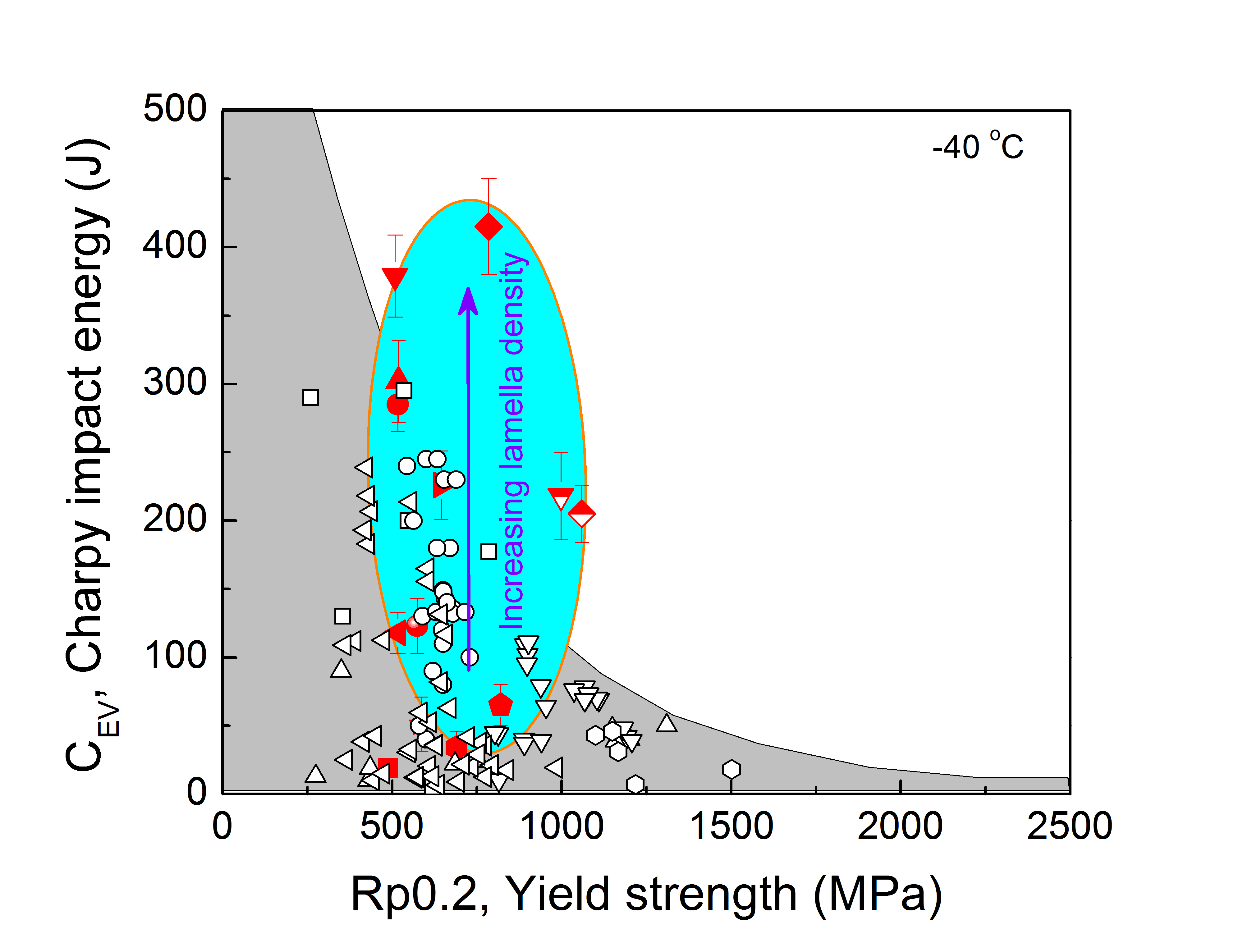
**

**Figure S4** Optical micrographs showing the microstructures produced in 5Mn3Al DP-steels after hot rolling: (a) 0.05C5Mn3Al, (b) 0.10C5Mn3Al, (c) 0.15C5Mn3Al, (d) 0.20C5Mn4Al, in which the phase with light grey color is ferrite lamella and the dark is martensite lamella. 1: Normal direction (ND) ; 2: Transverse direction (TD).


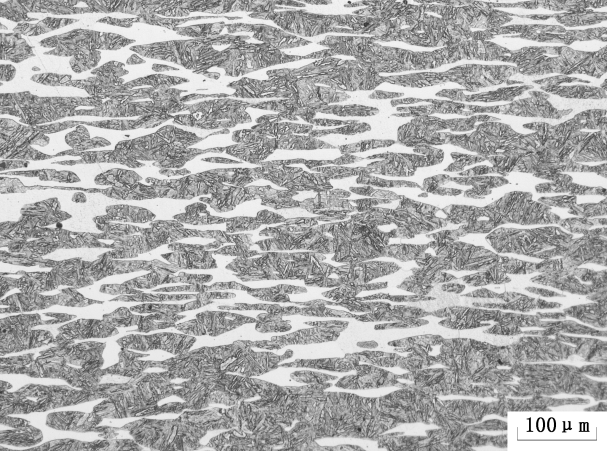

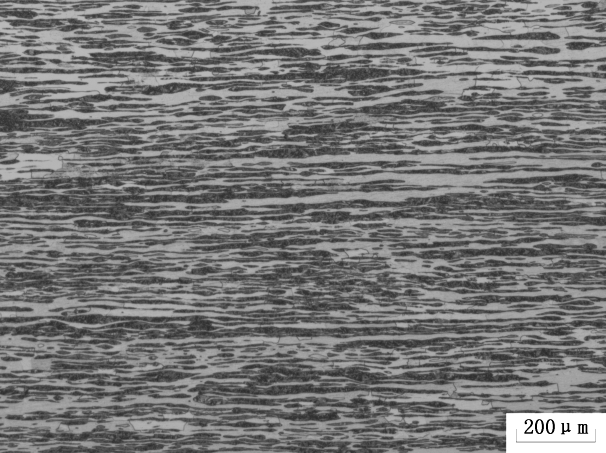

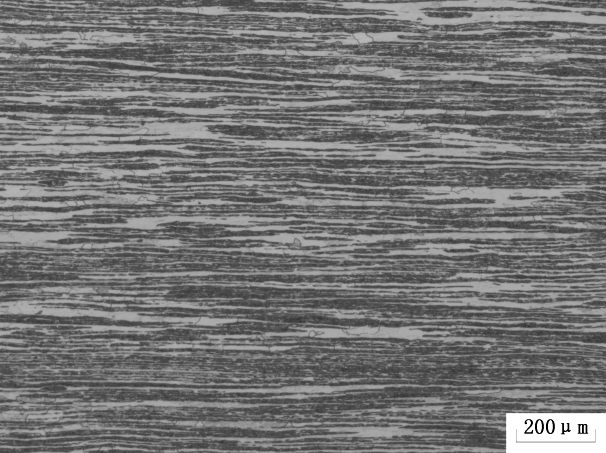

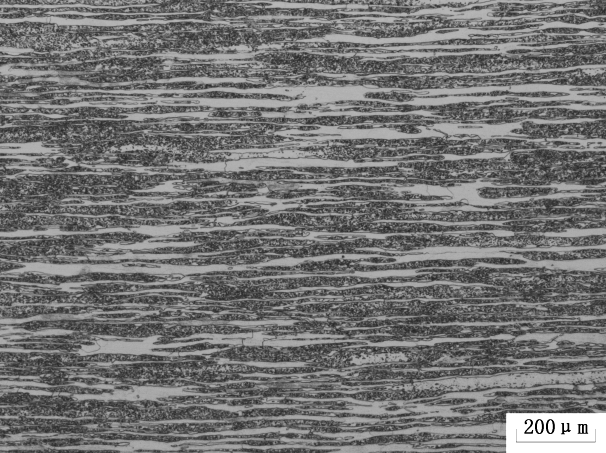

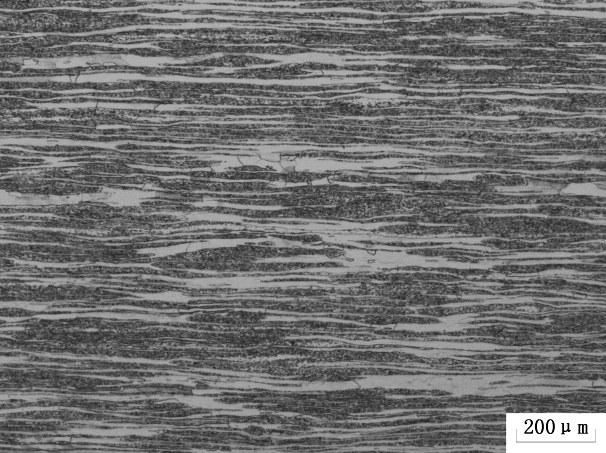

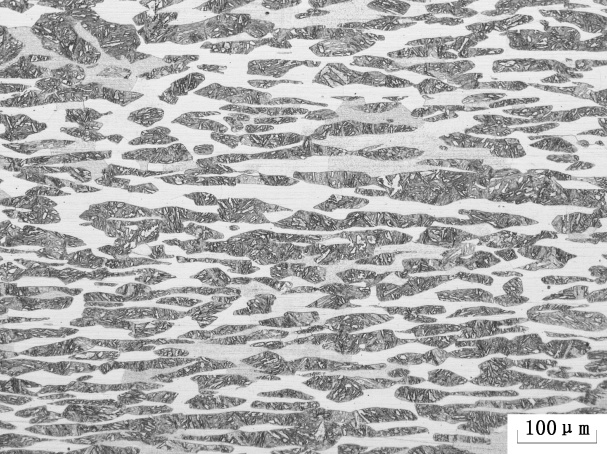

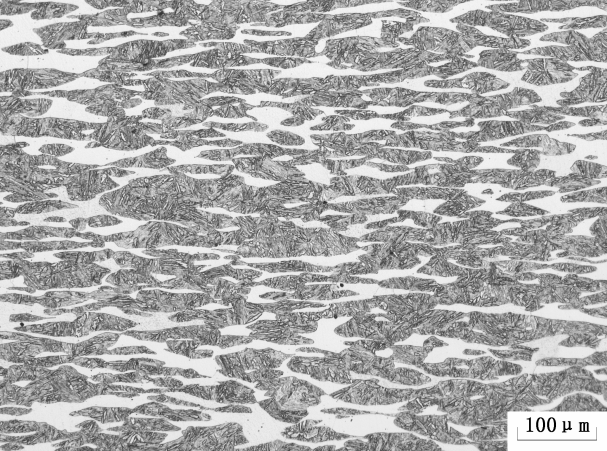

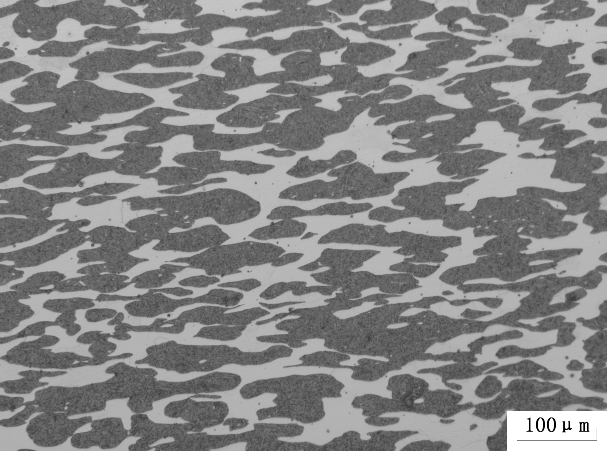


**RD**

**ND**

**TD**

**ND**

(a1)

(b1)

(c1)

(a2)

(d2)

(b2)

(c2)

(d1)

**Figure S5** Comparison of the diffusion coefficient of C, Mn and Al in α (bcc) and γ (fcc) phases in steel at given temperature range. It can be seen that in the range of 800-1200 ^o^C the value of C is significant higher than that of Al and Mn. (Data from: Brandes, E. A. Smithells metals reference book. 6 edition, london: butterworths, 1983)


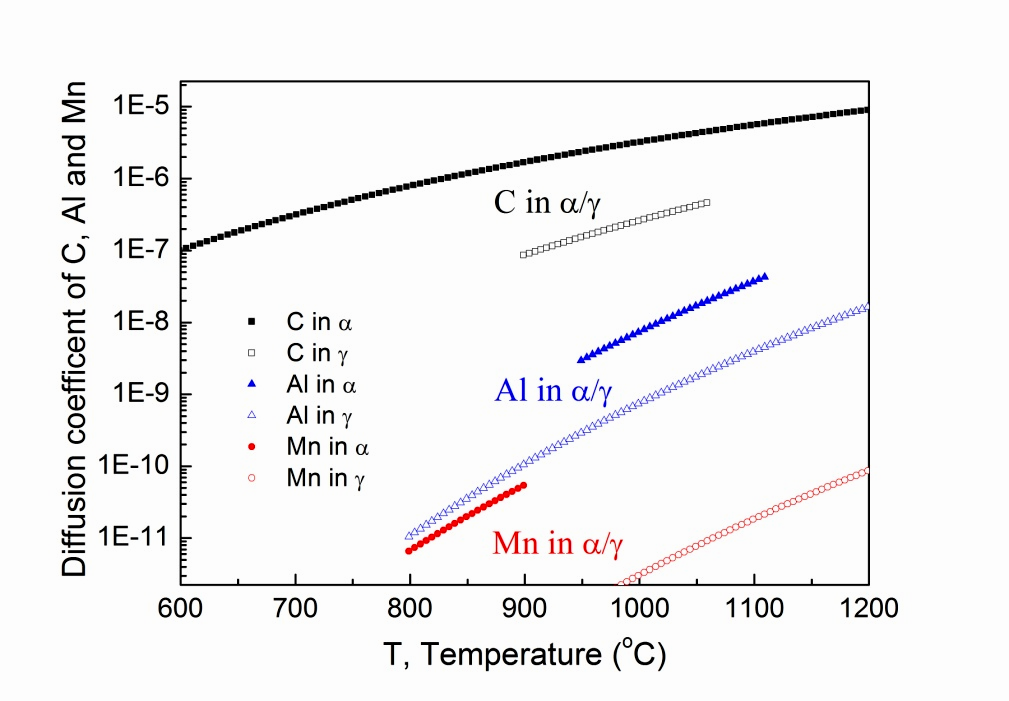

Supplement: Supplementary Meterials [file srep41459-s1.doc]
